# Conditional survival analysis in Korean patients with gastric cancer undergoing curative gastrectomy

**DOI:** 10.1186/s12885-015-2022-2

**Published:** 2015-12-23

**Authors:** Jin Won Lee, Bandar Ali, Han Mo Yoo, Cho Hyun Park, Kyo Young Song

**Affiliations:** Division of Gastrointestinal Surgery, Department of Surgery, Seoul St. Mary’s Hospital, College of Medicine, The Catholic University of Korea, 222 Banpo-daero, Seocho-gu, Seoul 137-701 South Korea

**Keywords:** Survival, Gastric cancer, Gastrectomy, Prognosis, Follow-up

## Abstract

**Background:**

Conditional survival (CS) measures the probability that patients will survive an additional number of years given that they have already survived for a certain period of time.

**Methods:**

In total, 2935 gastric cancer patients who had undergone curative gastrectomy between 1995 and 2011 were enrolled. The Cox proportional hazard regression model was used to evaluate the factors associated with overall survival (OS). Three-year CS estimates at ‘t’ years after surgery were calculated as follows: CS(t) = S(t + 3)/S(t).

**Results:**

The 1-, 2-, 3-, 4- and 5-year OS rates of the 2935 patients were 96.6 %, 92.0 %, 88.7 %, 85.6 and 82.7 %, respectively. The probability of surviving an additional 3 years on the condition of having already survived 1, 2, 3, 4 and 5 years after surgery were 88.6 %, 89.9 %, 91.0 %, 92.2 % and 93.2 %, respectively. Patients with a higher risk at baseline showed a greater increase in CS over time.

**Conclusions:**

CS estimates provide important information about dynamic prognostic changes over time for Korean gastric cancer patients, and as such, can be used to guide long-term follow-up strategies.

## Background

Despite declining global incidence rates, gastric cancer remains the fourth most common malignancy and the second leading cause of worldwide cancer-related mortality [1]. The cornerstone of curative therapy for gastric cancer remains surgical resection with adequate lymphadenectomy. In recent years, there have been many advances in treatment options, including the establishment of surgical techniques for tumor resection and lymph node dissection. Together with progress in adjuvant chemotherapy, radiotherapy, and molecular-targeted therapy, the long-term outcome of patients with gastric cancer has improved considerably. Therefore, to maximize the efficacy of treatment and follow-up strategy, it is important to establish the prognoses of individual patients and to apply suitable treatment strategies.

Previous comparisons of risk-grouping approaches in several diseases using nomograms have shown improved predictive accuracies. A further potential benefit of nomograms is that, through their simple graphical representation of a statistical predictive model, they generate a numerical probability of a clinical event [2].

In general, cancer patient survival is calculated from the day of operation to the most recent follow-up visit or death. Traditional survival estimation and initial prognosis assessment at the time of surgery facilitate adjuvant chemotherapy selection and follow-up scheduling. This approach, however, lacking postoperative follow-up information, provides only a static view of risk, because the prognosis of patients who have already survived for a certain period of time after their initial treatment is not the same as the initial prognosis [3, 4]. Furthermore, patients often ask questions regarding their probability of survival from the time they begin to visit a clinic for follow-ups, but physicians and clinicians are unable to adequately respond based only on the 5-year overall survival (OS) reported at the time of surgery.

Conditional survival (CS) estimates are based on the concept of conditional survival probability [5]. They represent the probability of surviving additional years conditional to having already survived for a certain period. Thus they account for changes in hazard rate over time as well as for dynamic changes in prognosis. The usefulness of CS estimates has been established for many solid malignancies including urothelial, colorectal, thyroid, ovary, breast, lung and gastric tumors [3, 4, 6–15]. A multicenter analysis by the US Gastric Cancer Collaborative indicated that CS estimates provided important information on changes in the probability of survival over time [16].

However, to date there has been no comparable study on Korean patients with gastric cancer. The aims of this study, therefore, were to estimate, based on the analysis of a large-scale database, the CS of Korean gastric cancer patients and to determine its usefulness to those patients’ prognoses.

## Methods

### Patients and data collection

In total, 2935 patients who had undergone curative radical gastrectomy at our hospital between January 1995 and December 2011 were included in this study. The relevant demographic, preoperative, postoperative, and pathologic data were collected from the patients’ medical records. Preoperative disease assessment was based on physical examinations, blood tests, chest and abdominal X-rays, endoscopy, abdominal computed tomography scans and positron emission scanning. Pathological staging was assigned using *The American Joint Committee on Cancer* (*AJCC*; 7^th^ ed.) [17], in which depth of invasion is defined by T1 tumor invasion of the mucosa and submucosa, T2 tumor invasion of the muscularis propria, T3 tumor penetration of the subserosal connective tissue without invasion of the visceral peritoneum, and T4 tumor invasion of the serosa (T4a) or adjacent organs (T4b). Data on recurrence and overall survival (OS) were collected. Approval for this study was obtained from the Institutional Review Board of Seoul St. Mary’s Hospital (KC15RI0252). The research was conducted in compliance with the Helsinki Declaration.

Only patients undergoing resection with curative intent were enrolled. Patients undergoing palliative resection and those with known metastatic disease, 30-day perioperative mortalities, and a history of other organ malignancies were excluded. For each patient, the following parameters were recorded: age, gender, performance status according to the Eastern Cooperative Oncology Group (ECOG) scale, tumor location, resection extent (total gastrectomy, subtotal gastrectomy and others), main-tumor size (cm), histologic type (differentiated type: well or moderately differentiated tubular and papillary adenocarcinoma; undifferentiated type: poorly differentiated adenocarcinoma, signet-ring cell or mucinous carcinoma), lymphatic invasion (LI), vascular invasion (VI), neural invasion (NI), Lauren classification (intestinal, diffuse or mixed type), tumor-node metastasis (TNM) stage, number of retrieved lymph nodes, and number of positive nodes.

### Conditional survival (CS) estimates

The primary outcome was 3-year CS (CS_3_) at each time point. CS is the estimation of the probability that a patient will survive for an additional number of years given that he or she has already survived a certain period of time [17]. The CS_3_ at ‘t’ years after surgery is defined as the probability of surviving an additional 3 years after t years. This was calculated as CS(t)(%) = [S(t + 3)/S(t)] × 100. The Kaplan-Meier log-rank test and the Cox proportional hazard regression model were used to evaluate the factors associated with OS. CS was compared with OS according to the variables determined by multivariate analysis to be independent risk factors for survival.

### Statistical analysis

Continuous data were expressed as means ± standard deviation. The independent factors significantly related to patient survival were evaluated with reference to statistics obtained by the Kaplan-Meier method for calculating OS, and the multivariate Cox proportional hazard model was utilized to assess the effects of the variables on OS. All of the tests were two-sided; statistical significance was predefined at *P* < 0.05. All of the statistical analyses were performed using SPSS software (version 12.0; SPSS, Chicago, IL, USA).

## Results

### Patient characteristics

The baseline characteristics of the 2935 patients are shown in Table 1. The mean age was 57.8 years, and 66 % (*n* = 1424) were male. The majority of patients had a performance status of 0 or 1. At the time of surgery, the great majority of patients underwent either a subtotal gastrectomy (75.4 %) or a total gastrectomy (24.0 %), with the remaining 0.6 % undergoing other operations (either Whipple’s procedure, proximal gastrectomy or wedge resection). The mean tumor size was 4.07 cm. With regard to the Lauren classification, 43.7 % of patients had an intestinal-type tumor, while the remaining tumors were either diffuse or of the mixed type. Most tumors were located in the lower third of the stomach (*n* = 1632, 55.6 %), and half of the tumors were early-stage cancer (T1 tumors: *n* = 1594, 54.5 %). Based on the *The AJCC* (7^th^ ed.) staging system, most of the patients had stage I tumors (*n* = 1743, 59.4 %); the remaining patients had either stage II (*n* = 553, 18.8 %) or stage III (*n* = 639, 21.8 %) tumors (Table 1).

**Table 1:** Clinicopathologic characteristics associated with survival in 2935 patients after curative gastrectomy for gastric cancer

	No (%)	5-YSR	P* value
Age			0.000
Mean ± SD	57.8 ± 11.95		
<60	1511 (51.5)	86.2	
≥60	1424 (48.5)	78.8	
Sex			0.303
Male	1931 (65.8)	82.0	
Female	1004 (34.2)	83.9	
ECOG PS			0.000
0	1272 (43.3)	90.6	
1	1535 (52.3)	78.2	
2	56 (1.9)	53.5	
3	4 (1)	50.0	
4	1 (0)	0	
5	0 (0)	-	
Unknown	67 (2.3)	85.0	
Resection extent			0.000
TG	704 (24.0)	73.3	
STG	2212 (75.4)	85.7	
Others	19 (0.6)	68.1	
Tumor size			0.000
Mean ± SD	4.07 ± 2.80		
<5 cm	2004 (68.3)	91.0	
≥5 cm	931 (31.7)	65.0	
LI			0.000
-	1784 (60.8)	94.5	
+	1130 (38.5)	64.3	
Unknown	16 (0.5)		
VI			0.000
-	2762 (94.1)	84.1	
+	141 (4.8)	53.5	
Unknown	26 (0.9)		
NI			0.000
-	2206 (75.2)	89.4	
+	697 (23.7)	62.2	
Unknown	25 (0.9)		
Leuran classification			0.000
Intestinal	1283 (43.7)	86.1	
Diffuse	993 (33.8)	79.9	
Mixed	601 (20.5)	69.4	
Unknown	58 (2.0)		
Histology			0.000
Differentiated	1358 (46.3)	85.5	
Undifferentiated	1577 (53.7)	80.1	
Location			0.000
Esophagus	34 (1.3)	72.3	
Upper 1/3	286 (9.7)	80.6	
Middle 1/3	947 (32.3)	83.2	
Lower 3	1632 (55.6)	82.9	
Duodenum	1 (0)	100	
Whole Stomach	11 (0.4)	53.3	
Unknown	24 (0.8)	100	
Depth			0.000
T1	1596 (54.4)	96.0	
T2	346 (11.8)	87.4	
T3	532 (18.1)	71.6	
T4a	442 (15.1)	47.7	
T4b	17 (0.5)	48.7	
Total number of			
Retrieved LN			
Mean ± SD	41.62 ± 16.27		
Total number of			
Positive LN			
Mean ± SD	2.56 ± 5.95		
Nodal status			0.000
N0	1887 (64.3)	94.3	
N1	366 (12.5)	79.8	
N2	308 (10.5)	71.2	
N3	373 (12.7)	39.3	
TNM Stage			0.000
I	1743 (59.4)	96.1	
II	553 (18.8)	80.9	
III	639 (21.8)	49.7	

Continuous variables are presented as the mean ± standard deviation

No, number of patients; 5-YSR, 5-year survival rate; *ECOG* Eastern cooperative oncology group, *PS* Performance status, *TG* Total gastrectomy, *STG* Subtotal gastrectomy, *LI* Lymphatic invasion, *VI* Venous invasion, *NI* Neural invasion, *LN* Lymph node, *TNM* Tumor-node-metastasis

^*^Log-rank test

### Prognostic factors associated with overall survival (OS)

Baseline demographic and tumor-characteristic variables associated with survival were selected for analysis. A univariate analysis using the Kaplan-Meier method identified all of the factors, except gender, as having a significant association with outcome. According to the multivariate analysis, age, performance status, tumor size, tumor depth, nodal status, and TNM stage were each independently associated with OS (all *P* < 0.05)(Table 2), which results are consistent with the findings of relevant previous studies [16, 18].

**Table 2:** Independent prognostic factors associated with time to death at multivariate analysis using cox proportional hazards model

	HR	95 % CI	*P* value
Age			0.000
<60	Ref	-	
≥60	1.760	1.489–2.079	
ECOG PS			0.001
0–1	Ref	-	
2–5	1.968	1.317–2.940	
Tumor size			0.014
<5 cm	Ref		
≥5 cm	1.267	1.050–1.529	
Depth			0.000
T1	Ref	-	
T2	1.499	1.097–2.050	
T3	2.160	1.640–2.845	
T4a	3.153	2.338–4.253	
T4b	4.910	2.504–9.628	
Nodal status			0.000
N0	Ref	-	
N1	1.151	0.820–1.616	
N2	1.379	0.979–1.942	
N3	2.863	2.062–3.976	
TNM Stage			0.000
I	Ref	-	
II	2.328	1.752–3.093	
III	3.387	2.235–5.133	

*HR* Hazard ratio, *CI* Confidence interval, *ECOG* Eastern cooperative oncology group, *PS* Performance status, *TNM* Tumor-node-metastasis

### Overall (OS) and conditional survival (CS) estimates

1. Overall patients
  Figure 1 shows that the OS decreased over time, whereas the CS_3_ increased. The OS at 3 years, 88.7 %, decreased to 80.7 % at 6 years. By contrast, the CS_3_ at 3 years, indicating the probability of surviving an additional 3 years postoperatively, was 91 %.

**Fig. 1:**
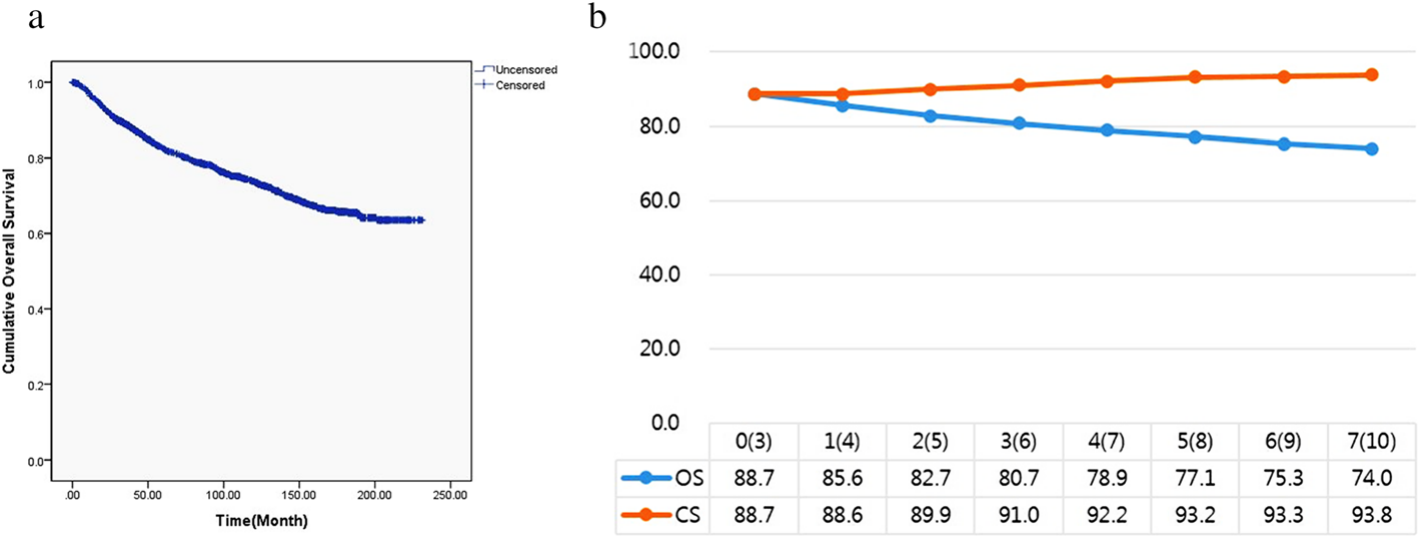
Survival analysis. **a** Overall survival (OS) curve of 2935 enrolled patients, **b** 3-year conditional survival (CS) relative to actuarial survival. The number in parentheses represents the exact OS time-point after surgery
2. High-risk patients
  The calculated CS_3_ for the strata of the prognostic factors found on multivariate analysis exceeded the OS, and the gaps between the OS and CS increased more prominently in patients with higher risk factors (i.e., those initially predicted to have a poor outcome) (Fig. 2). For example, the CS_3_ at 1- and 4-year OS in patients with a tumor size of 5 cm or more were 75.7 and 70.6 %, respectively. This difference between CS_3_ and OS increased, over time, to 36.7 % (CS_3_ at 7 years: 92.8 %; 10-year OS: 56.1 %) (Fig. 2a). Similarly, the CS_3_ at 7 years in patients diagnosed with N2 and N3 were 92.8 and 92.5 %, respectively, whereas the 10-year OS were 60.7 and 31.0 %, respectively (Fig. 2b). This trend was prominent also for the T stage (depth of invasion) and overall stage. For example, the CS_3_ of patients with T4b disease increased from 56.3 to 100 %. Finally, the CS_3_ in patients with stage III disease increased from 64.7 to 95.1 %, respectively (Fig. 2d). Interestingly, for patients who were at a more advanced disease stage, the CS_3_ was higher at the end of the study period.

**Fig. 2:**
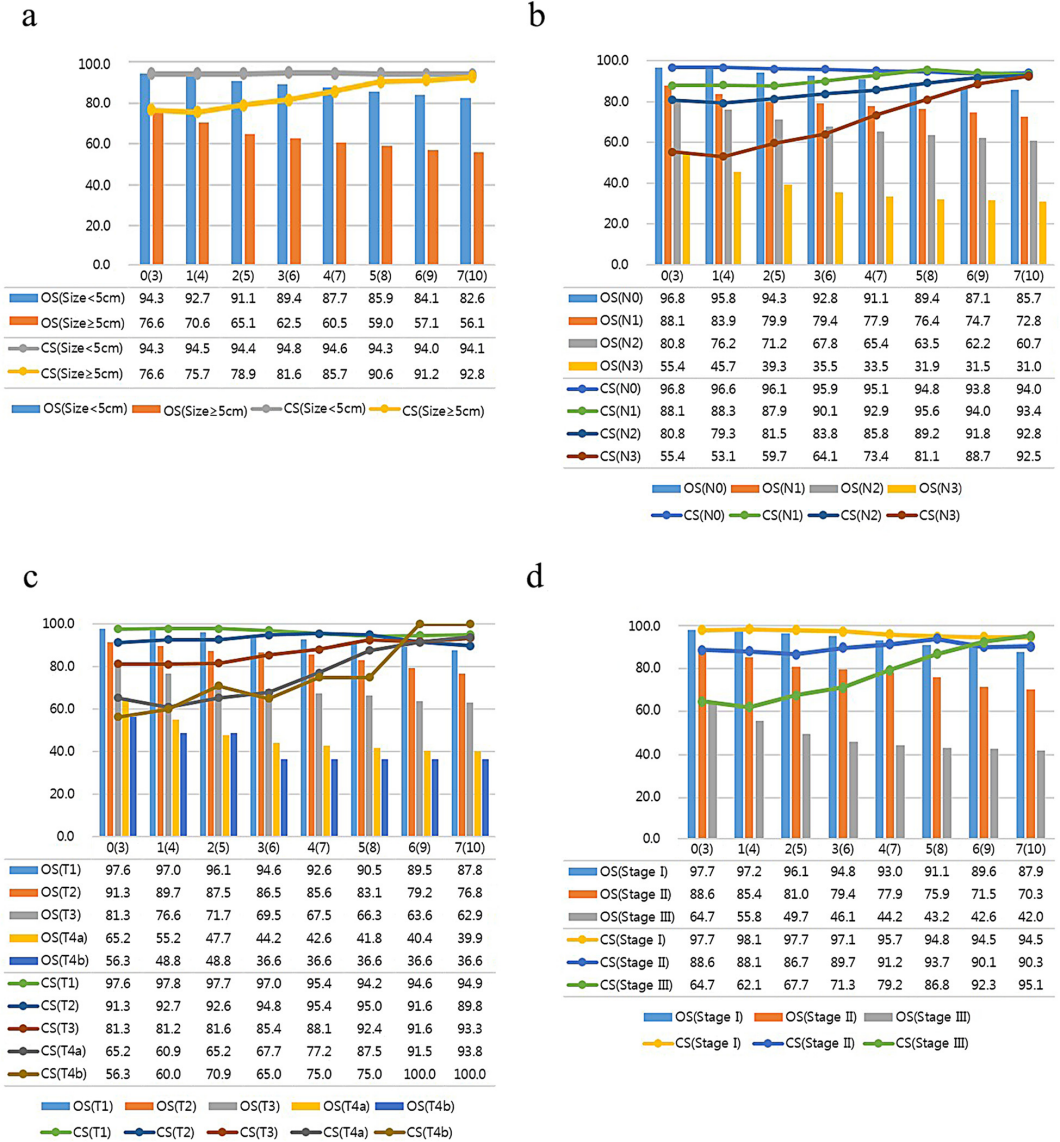
Comparison of OS with CS, stratified by **a** tumor size, **b** nodal status, **c** tumor depth, and **d** AJCC stage. The number in parentheses represents the exact OS time-point after surgery
3. Low-risk patients
  The CS_3_ at 1- and 4-year OS in patients with a tumor size less than 5 cm were 94.5 and 92.7 %, respectively, and the difference between CS_3_ and OS after 6 additional years increased only to 11.5 % (CS_3_ at 7 years: 94.1; 10 year OS: 82.6 %) (Fig. 2a) Furthermore, the CS_3_ at 7 years in patients diagnosed with N0 and N1 were 94.0 and 93.4 %, respectively, whereas the 10-year OS were 85.7 and 72.8 %, respectively (Fig. 2b). Lastly, the CS_3_ of patients with T1 disease decreased slightly from 97.6 to 94.9 % (Fig. 2c), and that of patients with stage I disease decreased similarly, from 97.7 to 94.5 % (Fig. 2d).

## Discussion

The prognosis of gastric cancer patients usually is evaluated in light of the postoperative pathological findings at the time of initial treatment [18]. In fact, the initial stage of gastric cancer is defined by the AJCC as one of its most important prognostic indices, based on tumor-associated factors such as extent of primary tumor (T), regional lymph nodes (N), and distant metastases (M) at the time of surgery, thus providing staged grouping based on T, N and M [17]. Thus far, many factors have been shown to be of prognostic significance to the 5-year survival rate. These include TNM stage, grade, primary tumor size, tumor location, lymphatic and vascular invasion, age, and gender [19]. As shown by multivariate analysis, age, performance status, TNM stage and tumor size are independent prognostic factors for survival. However, the prognosis of patients at the same stage is not completely homogeneous, and the risk-factor hazard function is not constant over time. Although prognostic predictions are useful for guiding the selection of the treatment modality, they might lose their accuracy once a patient passes the predicted milestone.

Our present results provide information pertinent to the conditional 3-year survival rates of patients with gastric cancer. In this study, as the postoperative time progressed, the next 3-year survival rates for patients in each group, stratified by various factors, not only improved with prolonged survival, but also became similar in value by the end of the study period. The OS rates decreased with time, whereas the CS_3_ estimates increased with time. Yoshihiro et al. [18] concluded that survival is not only stage dependent at the time of surgery but also dependent on the length of survival. They emphasized that prognoses at 1, 2, 3, 4 and 5 years after the initial operation differed from those estimated just after surgery, due to the uncertainty and heterogeneity of the characteristics of this disease at the time of surgery. Correspondingly, our findings suggest that the probability of patient survival evolves over time, and that the impacts of initial prognostic factors decrease with increasing survival [16, 18]. We found that CS increases markedly over time, especially in patients with late-stage disease.

The CS estimate, a novel prognostic index, provides new information relevant to the dynamic prognostic changes that occur for patients with gastric cancer. Several studies have shown that CS estimates are highly significant to the evaluation of the prognostic prospects of patients with various malignant diseases [4, 6–16, 20]. Furthermore, it is generally accepted that patients with poor prognostic features at the time of initial diagnosis show, compared with those without these features, greater increases in CS over time. Kim et al. [16] demonstrated that patients at higher risk at baseline showed the greatest increases in CS over time. In their study analyzing 807 patients who had undergone resection for gastric adenocarcinoma, the 1-, 3- and 5-year OS rates after gastric resection were 42, 34, and 30 %, respectively. The CS estimates, which is to say, the probabilities of surviving an additional 3 years given that the patient had already survived 1, 3, and 5 years, were 56, 71, and 82 %, respectively. Our present 1-, 3- and 5-year OS rates after initial surgery were much higher than Kim et al.’s: 96.6, 88.7 and 82.7 %, respectively. The discrepancies between the two cohorts probably are attributable to the fact that our study population included a relatively high proportion of patients diagnosed with early-stage disease at presentation. The CS estimates of our patients’ probabilities of surviving an additional 3 years given their initial 1-, 3- and 5-year survivals were 88.6, 91.0 and 93.2 %, respectively. The subsequent 3-year survival rates of patients with worse prognostic factors appeared to approximate, as time passed, those of patients without such risk factors, and the patients with poorer prognostic factors (i.e., tumor size ≥ 5 cm, T4b, N3, stage III) showed the greatest increases in CS over time. This is due to the fact that the death hazard in patients with gastric cancer is highest during the first few years after the initial surgery, and those who are expected to die based on the estimated 5-year OS typically experience disease recurrence and death during that period [8].

There is a critical limitation to this study. Although we enrolled relatively large numbers of patients, selection bias might have been occurred in diagnosis, treatment, and the follow-up schedule, due simply to the study’s retrospective nature. Also, comorbidity data, adjuvant chemotherapy treatment, socioeconomic status and educational level were not evaluated as factors in predicting survival.

In recent years, patients with gastric cancer have been able to survive longer than those diagnosed a few decades ago [1]. This is thanks partly to higher rates of early detection, the development of better chemotherapeutic agents, and improved postoperative care. More than 50 % of patients undergoing gastrectomy in Korea are diagnosed with early gastric cancer. This positive trend, however, has necessitated more accurate prognostic assessment for correspondingly enhanced treatment planning and follow-up strategies [8, 21]. Since many Korean gastric cancer patients have a good chance for long-term survival, CS estimation is even more important than for Western patient populations.

## Conclusions

In conclusion, we demonstrated that CS improves over time following resection for gastric cancer. The adoption of CS estimates can help surgeons and oncologists to better predict survival, make the most appropriate treatment decisions, and conduct a more fully informed discussion with patients in light of their survival expectancy or prognosis. This is very important, particularly as many patients are followed closely for years after surgery, and naturally, they desire to know how long they can expect to survive. For further work, the risk-factor hazard function, the impact of which on survival varies over time, should be evaluated. Moreover, detailed knowledge of time-dependent risk profiles is required for improved prediction of survival and better-individualized follow-up strategies.

## Consent

Approval for this study was obtained from the Institutional Review Board of Seoul St. Mary’s Hospital Written informed consent from the patient for the publication was waived because this report is retrospective study.

## Abbreviations

CS: Conditional survival
TNM: Tumor-node metastasis
AJCC: American joint committee on cancer
ECOG: Eastern cooperative oncology group
OS: Overall survival
LI: Lymphatic invasion
VI: Venous invasion
NI: Neural invasion
